# Surveying the Perspectives of Parents and Professionals on Providing Upright, Hands-Free, Self-Initiated Mobility to Children with Severe Physical and Communication Disabilities

**DOI:** 10.3390/children12081024

**Published:** 2025-08-04

**Authors:** Fei Luo, Sarah W. Blackstone, Jesse Canchola, Vicki Casella

**Affiliations:** 1 The Bridge School, Hillsborough, CA 94010, USA; 2 StatCon Consulting, Hayward, CA 94544, USA; jcanch@statcon.net

**Keywords:** severe physical and communication disabilities, augmentative and alternative communication, self-initiated mobility, hand-free support walkers, interprofessional collaborative practice

## Abstract

**Background/Objectives**: Children with severe physical and communication disabilities face many challenges. They have very limited opportunities for upright, hands-free, self-initiated mobility. Current findings in neuroscience and theories on child development suggest that self-initiated mobility can have positive cascading effects on various developmental areas, including language and communication. This study was conducted to examine the current use of hands-free support walkers with children who have severe physical and communication disabilities and use augmentative and alternative communication and to identify the benefits and problems perceived by their parents and professionals from different disciplines. **Methods**: Online surveys were utilized to collect information from 127 participants, including 31 parents and 96 professionals or paraprofessionals. **Results**: The participants reported that these children could perform various motor activities in the hands-free support walkers to achieve different goals. Benefits identified by both parents and professionals included providing a way to exercise and stay active, improving motor control, enhancing independence, and bringing enjoyment. Professionals also observed positive impacts on communication, vocalization, use of eye contact, and problem solving. **Conclusions**: Results suggest that children with severe physical and communication disabilities can benefit from the upright, hands-free, self-initiated mobility provided by hands-free support walkers. Clinical implications and needs for future research are discussed.

## 1. Introduction

Children with severe physical and communication disabilities confront a number of challenges. They may have difficulty using their upper and lower extremities. They often sit in wheelchairs or strollers for most of the day and are pushed or carried from place to place. They have limited opportunities for self-initiated mobility, which is “moving oneself by using the body or using a mobility device” [1]. These children may have limited speech and need to use augmentative and alternative communication (AAC) tools, methods and strategies (e.g., gestures, facial expressions, manual signs, photos, line drawings, drawings, writing, speech-generative devices, etc.) to communicate across partners and contexts. In addition, some of these children may have cortical/cerebral visual impairment (CVI), which is a brain-based visual impairment. Children with CVI can see but have difficulty interpreting what they see, which limits their access to moving, learning, language development, and social interaction, as well as using AAC tools and technologies. As a result, these children are at risk across developmental areas.

Because they face a myriad of difficulties, children with severe physical and communication disabilities typically receive services from multiple professionals, including special education teachers, speech–language pathologists (SLPs), physical therapists (PTs), occupational therapists (OTs), assistive technologists (ATs), teachers of visually impaired (TVIs), and sometimes, orientation and mobility (O&M) specialists. These professionals may work in different settings, including school-based settings (e.g., public schools, private schools, special education schools, etc.) or community-based settings (e.g., private practice, hospitals, community clinics, etc.). Possible intervention goals for these children often include improving gross and fine motor skills, functional mobility, activities of daily living, and language and communication skills, and for children with CVI, accommodations to support their use of vision.

In schools, professionals often provide services separately, working on goals in their own areas outside the classroom, and may be unaware of the goals of their colleagues [2,3,4,5,6]. Parents of these children and professionals often have limited opportunities for collaboration, as they may only come together during annual Individualized Education Planning (IEP) meetings. Some studies showed that parents’ participation in IEP meetings was minimal, and the IEP goals were often developed without their input [7,8]. Additionally, in Blackstone et al. [9], parents reported a lack of adequate guidance on how to effectively assist their children at home and within their communities. The limited collaboration among professionals as well as between professionals and parents can restrict the development of a cohesive, team-based approach to supporting a child’s need [10].

This current approach to providing services to children with severe physical and communication disabilities stands in contrast to best practices proposed by researchers and supported by theories on child development. Mandak & Light [11] stressed the importance of shared decision-making and regular communication between team members, while Ogletree [12,13] underscored the need for cohesive and consistent support strategies. McCarty & Miller [14] used a case example to highlight the benefits of sampling and integrating services across various contexts.

Similarly, Smith & Thelen [15] advocated to use the dynamic systems theory as a framework for understanding and addressing developmental challenges. Dynamic systems theory proposes that child development unfolds within the interplay of multiple systems (e.g., motor, sensory, language, etc.) and changes in one system can have significant impacts on other systems [15,16]. This theory suggests that changes occurring in the motor system can bring changes in other systems. A number of studies have demonstrated that acquisition of new motor skills such as grasping, sitting, crawling, and walking can create more and different opportunities for children to explore their environment, which brings cascading effects on the development of language, social interaction, spatial memory, vision, etc. [17,18,19,20,21,22]. The embodied cognitive theory also suggests that children’s cognitive skills, including language skills, are acquired through interacting with physical and social worlds [16,23,24]. The theories described above highlight the importance of providing children with physical access to explore and interact with objects and people around them. These experiences can reshape brain structure and function due to its plasticity [25,26,27,28]. These theories and findings support the idea that providing new motor skills to children with severe physical and communication disabilities—a change in the motor system—may improve their cognitive and language skills, which are one of the important intervention goals for these children and are essential for developing the use of AAC [29]. Upright, hands-free, self-initiated mobility can provide such opportunities for these children.

### 1.1. Upright, Hands-Free, Self-Initiated Mobility

In children with typical development, upright, hands-free, self-initiated mobility (i.e., walking) has a positive impact on various developmental areas through providing children with new ways to move, interact and explore objects and people at a distance, and see the environment from a new vantage point [20,30]. Walking has a positive impact on communication and language development. When children start to walk around 11 to 13 months, there is an increase in receptive and expressive language, regardless of age and culture [24,31,32]. Walking is also associated with increased use of gestures [33], vocalizations [34], and social interactions [35,36]. The onset of walking changes gaze patterns between children and caregivers [36,37]. For example, children who attain walking are more likely to look at their caregivers [36] and initiate eye contact [37]. Walking also changes caregivers’ behaviors, increasing their use of gestures and language [33,38]. Additionally, walking supports the development of spatial cognition [36] and spatial memory [39].

Children with severe physical disabilities can achieve self-initiated mobility using mobility devices, both powered (e.g., powered wheelchairs, battery-operated ride-on cars, robotic gait trainers) and non-powered (e.g., handheld walkers, support walkers with arm support, and hands-free support walkers). Studies on powered mobility devices for children with cerebral palsy (CP) and other developmental disabilities (e.g., spinal bifida) suggest that such devices foster independence, exploration, participation, and peer interaction while positively impacting cognition, language, communication, and social skills [1,40,41,42,43].

**Figure 1:**
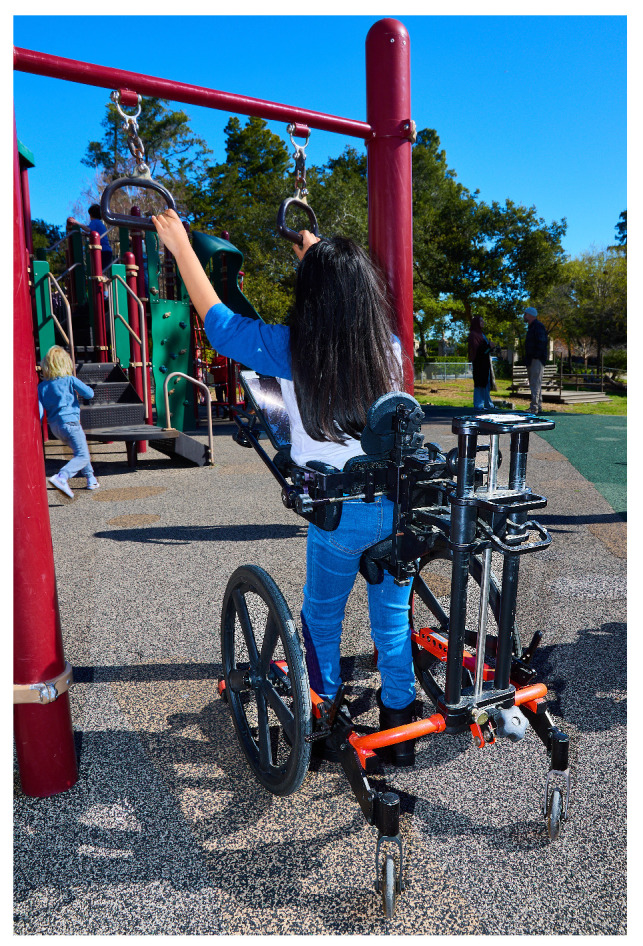
An example of a hands-free support walker.

Children with severe physical disabilities can achieve upright, hands-free, self-initiated mobility using a hands-free support walker (HFSW). The HFSW is a mobility device designed to provide support to a child’s pelvis and body using a seat and trunk pads, and a headrest when needed [44]. Figure 1 shows an example of the HFSW. Unlike handheld walkers and support walkers with arm support, the HFSW allows the child to have their hands free, providing opportunities for reaching, touching, and exploration. Various HFSWs, such as KidWalk, ProneWalk, Rifton Pacer, Grillo Ormesa, Mustang, offer different frame styles, wheel and caster options, and body support features [45]. The HFSW is typically used by children with CP classified at Levels IV or V on the Gross Motor Function Classification System (GMFCS) [46] with goals to foster independent mobility and participation [47]. The GMFCS is a five-level system that is used to classify children with CP according to their level of mobility and need for mobility devices, and Level IV or V suggests the need for extensive support in sitting, standing, and walking [47].

Research on the use of HFSWs with children with CP and other motor disabilities suggests that the upright, hands-free, self-initiated mobility provided by the HFSW can (a) increase independent mobility, physical activities, and hand function; (b) improve postural control, leg movement, and physical health; (c) promote independence; and (d) have a positive impact on affect, motivation, and participation [48,49,50,51]. Other noted benefits of the HFSW are gains in cognitive and motor development, social interaction opportunities, sensory motor experiences, quality of peer interaction, and a sense of self-worth [52,53,54]. McKeever et al. [55] also suggested that the HFSW provided more opportunities to participate in activities both at home and in the community, and children who used the HFSW were more socially accepted by siblings and peers with typical development. However, there is limited information on how the use of HFSWs impacts the development of language and communication.

The use of the HFSW can be affected by several factors that are both intrinsic to the child (e.g., growth, motivation, etc.) and extrinsic, such as access to different environments (e.g., types of the floor/ground surface, sizes of the space, etc.) as well as professional support [48,49,56]. Studies have identified some problems when using the HFSW, including the weight of the walker, the difficulty adjusting and repairing the walker, the difficulty transferring a child in and out of the walker, the need for adult supervision, and space issues [48,50,57,58]. In addition, as a child grows, it may become difficult to find a HFSW that fits, which is one of the reasons for discontinuing its use [57,59]. Other reasons may reflect changes in a child’s physical or health condition over time [52].

### 1.2. Upright, Hands-Free, Self-Initiated Mobility and AAC

Considering the theories and research findings described above, Luo and Blackstone [44] proposed that upright, hands-free, self-initiated mobility provided by the HFSW can positively influence the communicative competence of children with severe physical and communication disabilities who use AAC [60,61]. As described in Luo and Blackstone [44], upright, hands-free, self-initiated mobility may enhance social competence by enabling new behaviors for self-expression, such as moving towards objects to request or away to protest. It may support linguistic competence by creating language-rich learning opportunities, allowing children to explore, interact, and participate in new ways. Self-initiated mobility has been shown to improve spatial memory and spatial cognition [36,39], which may strengthen a child’s operational competence by their improved ability to locate symbols and navigate speech-generating devices effectively. Furthermore, strategic competence may develop as children gain opportunities to practice problem solving and self-initiated behaviors [62], which can foster the development of self-determination skills and adaptive strategies to overcome communication barriers [44].

Currently, there is limited information from parents and professionals on the use of HFSWs with children who have severe physical and communication disabilities and use AAC, including those with CVI. Research is needed to explore whether and how the use of HFSWs influence other developmental areas, particularly language and communication. Clinical observations suggest that upright, hands-free, self-initiated mobility provided by HFSWs positively impact the development of communication, language, and vision of these children [63].

The current study was conducted with two purposes. The first purpose was to identify how the HFSW was used in children with severe physical and communication disabilities. The second purpose was to examine the benefits and problems perceived by the parents of these children and professionals from different disciplines who worked with them. Given the limited collaboration typically observed between parents and professionals in schools [9,64,65], and parents obtaining the knowledge of their children in a unique way [66], parents and professionals may differ in their perception of using the HFSW. Therefore, differences between parents and professionals were also explored. Since there was limited research in this area, this study was designed as an exploratory study. Instead of testing specific hypothesis, the aim of the current study was to describe the perspectives of parents and professionals to inform future studies.

The following questions were specifically addressed: (a) How are HFSWs used with children with severe physical and communication disabilities? (b) What are the benefits related to the use of HFSWs? (c) What are the problems encountered when using HFSWs? (d) Do parents and professionals differ in their view of using the HFSW and the benefits and problems related to its use?

## 2. Materials and Methods

### 2.1. Participants

Two groups of participants were recruited nationally across the United States to participate in anonymous online surveys, i.e., parents and professionals. The parent group consisted of parents who had children with severe physical and communication disabilities, and the professional group included professionals (e.g., SLPs, OTs, PTs, etc.) and paraprofessionals such as instructional assistants, SLP assistants, etc., who worked with these children under the supervision of a licensed professional. Participants who submitted their surveys between November 2022 and April 2023 were included in the study. A total of 138 surveys were submitted and 11 were excluded because of incomplete data. Among the 127 remaining surveys, 31 were from parents and 96 were from professionals or paraprofessionals.

Parents reported living in 11 different states with 15 of them (50%; N = 30) in California, which likely reflects a regional concentration due to the outreach networks based in California. Three parents reported they were from other countries. To minimize participant burden and protect privacy, only the state of residence was collected from parents; no additional demographic information (e.g., age, education) was solicited. The mean age of the children was 11.8 ( SD = 8.3). Seventy percent of the parents (N = 30) reported their children were in an educational program, i.e., special education classes, home schooling, preschools, private schools, and regular education classes.

The parents reported the medical diagnoses of their children including cerebral palsy (74.19%), developmental delay (64.52%), complex communication needs (64.52%), epilepsy (48.39%), chromosomal abnormalities (16.13%), and muscular dystrophy (3.23%). About 23% of the parents reported other medical diagnoses including genetic diseases, deaf and blind, anoxic encephalopathy, and drug and alcohol exposure. They also reported the GMFCS level of their children, with 45.16% at level IV and 32.26% at level V. Among the participating parents, 90.00% (N = 30) reported that their children used AAC. The same percentage of parents reported that their children had a visual impairment. Among the different types of visual impairments reported, 88.89% (N = 27) had a diagnosis of CVI. Of the total parent participants, 83.87% reported their children were using, or in the past had used, a HFSW.

Professionals were from 18 states with 29 of them (37.66%; N = 77) from California. There were seven professionals from other countries. Professionals were from different disciplines. See Table 1 for number and percentage of professional participants from different disciplines. Professionals provided their services in different settings with 10–40% providing services in public schools, special education schools, clients’ homes, community therapy clinics, private schools, rehabilitation centers. Less than 6% provided services in state agencies, hospitals, school for the blind, community centers, private practice clinics, libraries, and charter schools. Among these professionals, 89 reported their work experiences, with 68.54% practicing more than 11 years. Of the total professional participants, 89.58% reported that their children were using, or in the past had used, a HFSW.

Professionals provided information about their caseload. Most worked with school age children with medical diagnoses that included cerebral palsy (89.89%), developmental delay (82.02%), complex communication needs (76.40%), chromosomal abnormalities (74.16%), epilepsy (53.93%), Rett’s syndrome (34.83%), muscular dystrophy (31.46%), spinal bifida (30.34%), and spinal cord injury (24.72%). About 10% of professionals reported other medical diagnoses, such as traumatic brain injury (TBI), autism spectrum disorder (ASD), alcohol and drug exposure, arthrogryposis, and genetic disease. They reported the GMFCS level of their clients with 31.82% at level IV and 35.23% at level V. Most professional participants (93.83%) reported that their clients used AAC; 90.12% reported their clients had a visual impairment with 97.22% reporting CVI.

The professional participants were asked if they were working with children who had a HFSW at the time of the survey. Among the 80 participants who responded to this question, 64 (80%) were working with children who used a HFSW, including 20 PTs, nine SLPs, nine special education teachers, eight TVI/O&M, seven ATs, five OTs, and six paraprofessionals. Over the past 10 years, 18.87% of these professionals had worked with one to five clients who used a HFSW; 28.30% worked with six to 10 clients; 16.98% worked with 11 to 20 clients; and 35.85% worked with more than 20 clients. Only these participants were asked to respond to more specific questions regarding the HFSW, specifically Questions 17 to 25 on the professional survey.

### 2.2. Reserch Design

Survey methodology was adopted to obtain information on how the HFSW was used with children with severe physical and communication disabilities and what benefits and problems perceived by their parents and professionals who worked with them. Because information in this area is limited, this is an exploratory study. We employed a non-probability convenience sampling method due to the unavailability of a complete sampling frame for the defined population. This approach is commonly utilized when it is impractical to identify or access all potential participants, making probability sampling methods infeasible [67]. Ethical approval for the study was obtained from North Star Review Board, an independent institutional review board.

No a priori power analysis was conducted because published prevalence estimates for this population were unavailable; however, a post hoc sensitivity check (G*Power 3.1) [68] showed that the achieved sample (N = 127) gives 80% power to detect effect sizes of Cramér’s V ≥ 0.25 at *α* = 0.05.

### 2.3. Materials

Three surveys were created to gather information needed for this study, i.e., an initial survey, survey for parents or care providers, and survey for professionals or paraprofessionals. See Supplemental Materials, Supplementary Survey A, B, C for the surveys.

A collaborative and iterative process was used to develop the surveys. The steps were as follows. First, questions and response choices were written by professionals who had extensive experiences supporting children with severe physical and communication disabilities who used HFSWs. Second, the first and second authors reviewed and refined the questions and response choices based on the previous studies on HFSWs. Third, a multidisciplinary research council including professionals and parents reviewed the refined questions and response choices. The questions and response choices were then revised accordingly. Fourth, REDCap was used to create the online version of the surveys. REDCap is an electronic data capture tool which is hosted by the Vanderbilt University Data Core Services [69]. Next, the online surveys were sent out to a small group of professionals and parents for testing. The feedback obtained from the testing was used to further refine the surveys. Lastly, the research team finalized the questions and responses choices, which were reviewed and approved by the research council. On the final surveys, most of the questions were close-ended so specific information could be obtained.

There were nine items on the initial survey: six single-response and three multiple-response (see Supplemental Materials, Supplementary Survey A). For example, Question 1 “Are you a parent/care provider or a professional/paraprofessional?” was a single-response item. Question 7 “For what reason(s) might your child not be using a hands-free support walker?” was a multiple-response item.

There were 33 items on the parent survey: 14 single-response, 17 multiple-response, and two written-response (see Supplemental Materials, Supplementary Survey B). For example, Question 4 “Please indicate the type of the educational program.” was a single-response item. Question 27 “Where does your child use the hands-free support walker?” was a multiple-response item. Question 23 “How old was your child when he/she first used a hands-free support walker?” was a written-response item.

There were 33 items on the professional survey: 9 single-response, 21 multiple-response, and three slider scales (see Supplemental Materials, Supplementary Survey C). For example, Question 3 “How many years have you been a practicing professional?” was a single-response item. Question 30 “What are the benefits you have observed from your clients when using hands-free support walkers? Select all that apply.” was a multiple-response item. Question 22 “What percentage of your clients who currently use hands-free support walkers wear Ankle Foot Orthoses (AFOs), which start at the toes and go to the upper calf?” was a slider scale item. Questions 17 to 25 were only answered by professionals whose clients were using the HFSW at the time of the survey, specifically, responding “yes” to Question 7, i.e., “Are you currently working with children who use hands-free support walkers?”

### 2.4. Procedures

#### 2.4.1. Survey Distribution

The surveys were distributed through the following three methods: (a) a public link to the survey was posted on websites and social media that parents and professionals who had children with severe physical and communication disabilities were likely to visit (e.g., The Bridge School website and social media, CVI/AAC Research Facebook Group, etc.); (b) a flyer containing the public link to the survey were sent out through mailing lists and at conferences and clinics (e.g., the American Speech–Language–Hearing Association Convention, California Children’s Services, etc.); and (c) an email invitation containing the public link to the survey was sent to parents and professionals whose email addresses were available in The Bridge School contact management database. A total of 4161 emails were sent. No information was available on which method a participant used to access the survey. At the beginning of the survey, a consent form was provided. The potential participants were informed that their participation was voluntary, and responses were anonymous.

#### 2.4.2. Data Collection and Analysis

The responses from the participants were collected and stored in REDCap. Data were exported for analysis in R [70] and SAS v9.4 [71]. Because the survey distribution list did not differentiate parents from professionals, role-specific response rates could not be calculated retrospectively. Because the participants were not required to answer all the questions, the sample size varied across different items in the surveys. Therefore, percentages were used for the data analysis. The percentage was calculated using the total number of participants who responded to a given question.

All statistical hypothesis testing used a 0.05 significance level. For each parent-versus-professional comparison, effect size was summarized using Cramér’s V [72], interpreted as small (~0.1), medium (~0.3), or large (~0.5). Three types of data were collected: (1) categorical, (2) quantitative, and (3) open-ended text responses.

For the categorical data type (e.g., state of residence, disciplines, medical diagnoses), standard frequency distributions were employed for univariate variables and RxC contingency tables were for cross-tabulated multi-dimensional data. To address the issue of multiplicity in this exploratory study, the false discovery rate (FDR) method was utilized to examine differences between groups. Particularly suitable for exploratory research, the FDR method was chosen over more conservative approaches as it allows for the capture of a *p*-value-controlled maximum set of significant findings or near significant findings that may advise further study.

For the quantitative data type (e.g., age of the child), descriptive statistics were used including the mean, median, standard deviation, minimum and maximum.

The third type of data included open-ended text responses from participants in a response to the “Other” category. These data were analyzed using the following steps. First, responses were reviewed to see if they could be re-coded using existing categories. Second, if a response did not belong to the existing categories, two researchers (i.e., first and second author) reviewed the responses to identify general categories independently. Third, the two researchers discussed the new categories until they reached 100% agreement.

## 3. Results

As part of the initial questions, all participants were asked if they believed that children with severe physical and communication disabilities could benefit from self-initiated mobility experiences provided by HFSWs. A majority (98.39%) provided a positive response, with over 85% reporting HFSWs could have a positive impact on multiple areas including physical, social, communication, self-determination, participation, cognitive, emotional, spatial, and visual areas. After the initial nine questions, the participants were directed to questions corresponding to their roles (parents or professionals). Not all results obtained from the surveys were reported in this section. The results that addressed the current research questions were presented, including the use of AAC, the use of the HFSW, and the benefits and problems of using the HFSW. Comparisons were also made between the responses from professionals (i.e., the professional group) and parents (i.e., the parent group) to explore the possible differences existing between the two groups.

### 3.1. The Use of AAC

**Figure 2:**
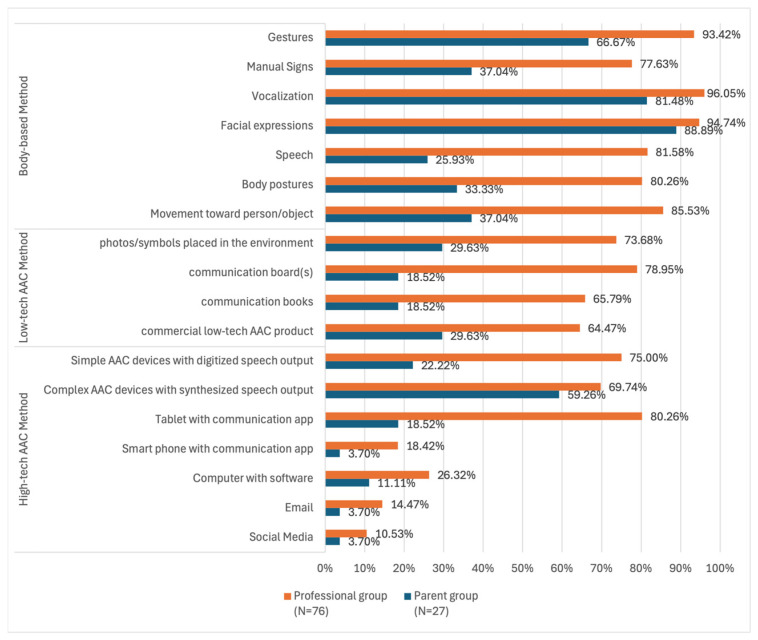
AAC methods indicated by the parents and professional groups.

The participants in both groups were asked if their children or clients used AAC. If the participants answered “yes”, they were asked to indicate the types of AAC methods used. The participants were not asked to specify if the children or clients used AAC when they were in the HFSW. Figure 2 shows the different types of AAC methods reported by both groups. Of note was that half of the parent group reported only four methods, i.e., facial expressions, vocalization, gestures, and complex AAC devices (e.g., Tobii). In contrast, 65% of the professional group reported using 14 out of 18 AAC methods listed.

### 3.2. The Use of the HFSW

Both groups of participants were asked to report how the HFSW was used, including the types of the HFSW used by their children or clients, the motor activities they could perform, and the purposes for using the HFSW. All participants in the parent group (N = 26) were given these questions. In the professional group, only participants whose clients were using the HFSW at the time of the survey were given these questions (N = 64).

#### 3.2.1. Types of the HFSW

For the different types of HFSWs reported by both groups, see Supplemental Materials, Supplementary Table S1. In the parent group, 22 participants reported the type of HFSW that their children used. The most common types were KidWalk (54.55%) and Rifton Pacer (18.18%). About 32% of parents selected “Other” and provided additional types of HFSWs not listed on the survey such as the Pony, R82 Crocodile, etc., or indicated that they had no knowledge of the type of HFSW that their children used. Five types of HFSWs listed on the survey were not reported by any parents.

In the professional group, 53 participants reported the type of HFSW that their clients used. KidWalk (75.47%) and Rifton Pacer (69.81%) were also the most common ones, followed by Mustang R82 (37.74%) and Grillo Ormesa (30.19%). No professionals reported using the Winnipeg/Pommel walker.

#### 3.2.2. Motor Activities Observed

**Figure 3:**
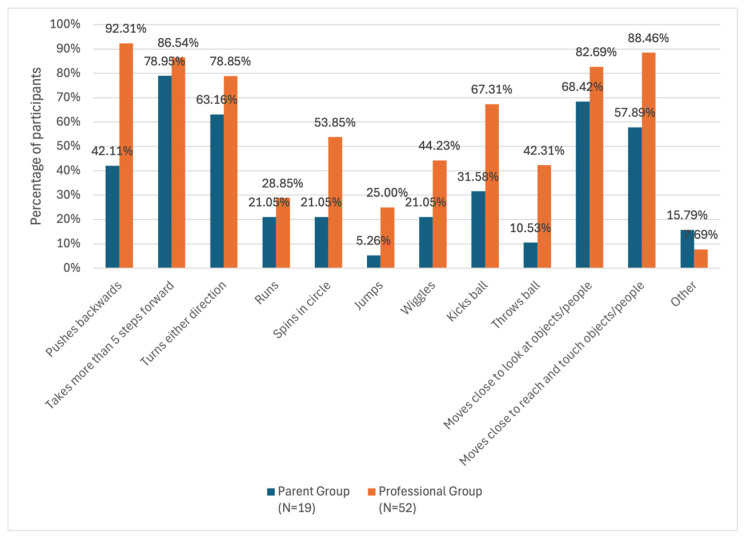
Types of motor activities observed by the parent and professional groups.

The participants in both groups reported the motor activities that their children or clients performed in the HFSW (See Figure 3). In the parent group, there were four motor activities observed by more than 50% of participants, including (a) taking more than five steps forwards; (b) moving close to look at objects or people; (c) turning either direction; and (d) moving close to reach and touch objects or people. In the professional group, there were seven motor activities observed by more than 50% of participants. In addition to the four motor activities mentioned above, professionals also reported pushing backwards, spinning in circles, and kicking a ball.

Comparisons were made between the groups using the FDR (i.e., false discovery rate). Compared to parents, significantly more professionals reported the following motor activities: (a) pushing backwards (*p* ≤ 0.0001); (b) spinning in circles (*p* = 0.033); (c) kicking a ball (*p* = 0.028); (d) throwing a ball (*p* = 0.033); and (e) moving close to reach and touch objects or people (*p* = 0.025). Cramér’s V indicated a large effect size for pushing back, a medium effect size for moving close to reach and touch, and a small to medium effect size for the rest of these activities. See Supplemental Materials, Supplementary Table S1 for the complete result of the comparison.

#### 3.2.3. Purposes for Using the HFSW

The participants in both groups were asked about the purpose of using the HFSW (See Table 2). Purposes were categorized into three groups, i.e., to achieve specific motor/mobility goals, to achieve ADL/functional goals, and to achieve educational goals. Among the top five goals reported, four were reported by both groups. Two of these goals were motor/mobility goals (i.e., to provide independent mobility and to improve muscle strength) and two were educational goals (i.e., to explore surroundings and to increase opportunities to participate with peers). The parent group also reported increasing activity levels as one of the top five goals, while the professional group reported accessing the school environment.

Comparisons were made between the groups with multiplicity adjustments to the *p*-value using the FDR method. Compared to the parent group, significantly more professionals reported using the HFSW to increase independence and participation in daily life (*p* = 0.044), access school environment (*p* = 0.013), and move to a specific location (*p* = 0.012). Cramér’s V indicated a medium effect size for these goals. Five goals were trending towards statistical significance: (a) to provide independent mobility (*p* = 0.078); (b) to participate in recess activities (*p* = 0.078); (c) to increase opportunities to participate with peers (*p* = 0.078); (d) to move close to see objects and people (*p* = 0.053); and (e) to access recess activities such as running, jumping, and playground games (*p* = 0.078).

### 3.3. Benefits of Using the HFSW

Participants in the parent (N = 26) and professional (N = 86) groups were asked about the possible benefits of using HFSWs in the following areas: (a) physical, (b) social/emotional/participation, and (c) other developmental areas (e.g., communication, vision, etc.) (See Table 3). The top five benefits selected by both groups included three physical benefits and two social/emotional/participation benefits. Four benefits were selected by both groups: (a) achieves a means for exercising and being physically active; (b) provides a change in position; (c) brings enjoyment; and (d) increases independence and access to surroundings. In addition to these four benefits, the parent group selected improving motor control while the professional group selected increasing opportunities for self-initiated mobility.

Comparisons were made between the groups. All professionals reported some benefits were observed while 9.52% of parents reported no benefits observed, which was significantly different (*p* = 0.037). Cramér’s V indicated a small-to-medium effect size.

Compared to the parent group, significantly more professionals reported the following physical benefits: (a) increasing opportunities for self-initiated mobility (*p* = 0.007); (b) enabling a child to move close to view objects and people (*p* = 0.037); (c) enabling a child to move towards a desirable place and move away from undesirable place or event (*p* = 0.037); and (d) acquiring new motor skills like jumping, wiggling, running, and spinning (*p* = 0.037). Significantly more professionals also reported benefits in other key developmental areas, including improving communication (*p* = 0.037), increasing verbalization/vocalization while standing and moving (*p* = 0.037), demonstrating more eye contact with people or objects (*p* = 0.050), and demonstrating curiosity and problem solving (*p* = 0.037). Cramér’s V indicated a small-to-medium effect size for these benefits except for increasing opportunities for self-initiated mobility. Cramér’s V for this goal indicated a medium effect size.

**Figure 4:**
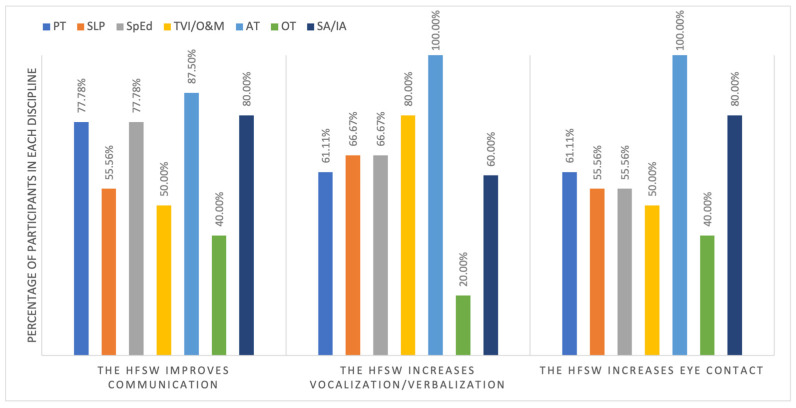
Benefits related to communication reported by professionals from different disciplines.

There were three benefits specifically related to communication, i.e., improving communication, increasing verbalization/vocalizations, and demonstrating more eye contact. A closer look was taken to examine the opinions of professionals from different disciplines on these three benefits. Figure 4 shows the percentage of professionals from different disciplines who selected these benefits. Although no statistical analyses were conducted, it appeared that more ATs reported these three benefits while fewer OTs did, particularly the benefit related to vocalization and verbalization.

### 3.4. Difficulities Encountered

The participants from the parent (N = 26) and professional (N = 86) groups were asked to identify the difficulties that they encountered when using the HFSW (See Table 4). The top two difficulties encountered by both groups were (a) not enough space indoors for the HFSW and (b) difficulty moving over uneven surfaces. The parent group also reported difficulty transferring in/out of the walker, while the professional group reported difficulty with the time and staffing required to transfer a client in/out of the walker. Comparisons were made between the groups, and no significant differences were found.

## 4. Discussion

Participants in the study were either parents of children with severe physical and communication disabilities or professionals working with them. Survey results provided insights about HFSW usage, highlighted benefits and challenges, and demonstrated differences in perspectives between parents and professionals. The discussion summarizes these findings and outlines future research to enhance HFSW and AAC design, use, and implementation across settings.

### 4.1. Use of AAC

A majority of participants from both groups reported that the children used AAC and had a diagnosis of CVI. Over 65% of parents indicated that their children used body-based AAC methods, such as facial expressions, vocalizations, and gestures. This finding aligns with studies such as Blackstone et al. [9], which highlight the reliance on body-based communication by children with CVI who use AAC. Similarly, Berenguer et al. [73] conducted a systematic review of parents’ perceptions and experiences with children with complex needs who used AAC, finding that many parents preferred to use body-based communication due to its ease and speed. Doak [74] observed that parents of children with autism spectrum disorder who used AAC often relied on body-based AAC methods, such as vocalizations, gestures, eye gaze, facial expressions, and even common household objects like shoes and doors. Parents valued these methods for their efficiency and intimacy, though they also recognized the limitations these methods posed regarding how, where, and with whom their children could communicate. In contrast, professionals reported the children they worked with utilized a broader range of AAC methods, including body-based, low-tech, and high-tech AAC.

This difference raises important questions about the availability of aided AAC tools or technologies in the home, the adequacy of parental training in using a range of aided AAC tools, and whether available AAC tools and technologies meet the needs of these children. A lack of knowledge, insufficient training and resources, limited communication and collaboration among stakeholders, and difficulties integrating AAC tools into daily routines have been identified as barriers to using aided AAC at home [73,75]. In addition, there remains a lack of research and guidelines on designing aided AAC systems tailored for children with severe physical and communication disabilities who also have CVI [76]. In the current study, over 80% of participants in both groups reported CVI. In other studies, the co-occurrence of CVI and other disabilities such as cerebral palsy have been found to range from 28% to 73% [77,78,79]. Parents have frequently reported that their children struggle to understand and use the language symbols commonly embedded in AAC systems, while professionals face challenges in recommending appropriate AAC tools for these children [9,65]. Additionally, none of the previous studies have considered how children with severe physical and communication disabilities access and use AAC tools and technologies when they are in the HFSW.

### 4.2. Use of the HFSW

#### 4.2.1. Types of the HFSW

In this study, KidWalk was the most frequently used HFSW reported by both groups. The second most commonly used HFSW was the Rifton Pacer. This finding contrasts with previous studies, such as those of Low et al. [56] and George et al. [52], in which the Rifton Pacer was the most commonly used HFSW. A systematic review by Livingstone & Paleg [50] identified the David Hart walker orthosis (later known as the Norsk Funktion-walker orthosis) as the most frequently reported HFSW in studies they reviewed. Notably, device selection is often influenced by factors such as geographic location, availability, cost, and funding resources [50]. This difference revealed by the current study suggests the limitation of the previous studies on the HFSW.

#### 4.2.2. Motor Activities Observed

Participants from both groups observed the children with severe physical and communication disabilities performed various motor activities in the HFSW such as taking steps forward, turning, and moving closer to look at or reach and touch objects/people. These motor activities can provide opportunities for exploration and interaction with objects and people. In addition, more than half of the professionals also observed children pushing backwards, spinning, and kicking a ball, which are new experiences for children with severe physical and communication disabilities. These new opportunities and experiences can have positive impact on language and communication development as suggested by the dynamic systems theory and embodied cognitive theory [15,16,17,18,19,20,21,22,23,24]. No previous studies have documented the different motor activities that can be performed by children with severe physical and communication disabilities when they are in the HFSW. Some studies have documented walking distance, endurance, speed, steering ability, and mobility independence [50].

#### 4.2.3. Purposes for Using the HFSW

Previous studies have highlighted the HFSW’s role in increasing mobility, physical activity, participation, and peer acceptance [48,52,55,80]. In this study, both parents and professionals identified similar goals, such as (a) providing independent mobility; (b) increasing activity levels; (c) enhancing independence and participation in daily life, recess and in inclusive physical education; and (d) expanding opportunities to engage with peers.

Notable differences were observed between the parents and professional groups. For instance, significantly more professionals reported using the HFSW to foster independence and participation in daily life, to access the school environment or to move to specific locations. In general, professionals reported more goals than parents. These findings suggest that professionals used the HFSW to address a broader array of goals compared to parents. The reasons for this discrepancy are unclear, but one possibility is that parents may not be fully aware of therapy goals. Limited collaboration and communication between professionals and parents, a challenge documented in other studies involving children with severe physical and communication disabilities who use AAC and have CVI [5,9,65], could be a contributing factor. Family involvement remains critical in interventions for children receiving speech–language therapy and those with complex needs [81,82,83].

### 4.3. Benefits of Using the HFSW

Participants in both groups identified several benefits of using the HFSW for children with severe physical and communication disabilities. Both parents and professionals reported that the HFSW helped children exercise, stay active, improve motor control, provide a change in position, gain more independence and access, and bring enjoyment. These findings are consistent with other studies showing that the use of HFSW contributes to improvements in physical health, physical activity, participation, and emotional well-being [50,52,55,56,80].

Both groups also reported benefits in other developmental areas, including cognition, communication, and vision. This finding supports the dynamic systems theory and embodied language theory, which suggest the change in one system—such as motor system—can have a cascading effect on other systems including cognition, language, and vision [15,16,19,21,22].

#### 4.3.1. Benefits Related to Language and Communication

There were three benefits on the survey related to language and communication, i.e., improving communication, increasing verbalization/vocalization, and demonstrates more eye contact. These observations align with findings observed in children with typical development, which demonstrate the cascading effects of upright, hands-free, self-initiated mobility (i.e., walking) on language and communication [24,31,32,34,36]. However, to date, no research has specifically explored the impact of upright, hand-free, self-initiated mobility on communication and language development of children with severe physical and communication disabilities who use AAC. Despite this gap, more than half of the professionals in this study reported observing these benefits.

Some differences were noted between parents and professionals. Significantly fewer parents reported these three benefits. Notably, close to 10% of parents reported not observing any benefits, a proportion significantly higher than professionals. Because of the current research design, what caused the difference cannot be identified. However, this finding is consistent with previous research showing differences in perspectives between parents and professionals that stem from their distinct relationships with the children, varying priorities, and a lack of mutual understanding [66,84]. It might also suggest that parents may require additional explanation and training to fully understand the benefits of upright, hands-free, self-initiated mobility and how the use of HFSWs might positively influence multiple developmental areas, including language and communication.

When examining these benefits reported by professionals more closely, some differences emerged among professionals from various disciplines. This suggests that certain disciplines may have a greater awareness of the benefits of upright, hands-free, self-initiated mobility than others. These findings underscore the importance of interdisciplinary collaboration to fully leverage the potential benefits of HFSWs for these children.

#### 4.3.2. Benefits Related to Vision

There were two benefits on the survey related to vision, i.e., using movement in a HFSW to attain and sustain vision and using vision to kick a ball, look for feet, reach for a favorite object, etc. Only about 50% of professionals and 30% of parents observed these benefits. The result indicates that the relationship between self-initiated mobility and vision in children with severe physical and communication disabilities who use AAC may not be well understood even though these children often experience visual impairments including CVI.

Studies have suggested that self-initiated mobility and vision can impact each other. On one hand, walking in children with typical development facilitates vision development because vision is developed through interacting with physical environment [18,85,86]. Improved vision can support the development of language and communication [87]. On the other hand, impaired vision may lower a child’s motivation to move, limiting opportunities for self-initiated mobility and interaction with objects and people in the environment [88,89]. This relationship highlights the importance of providing self-initiated mobility to children with severe physical and communication disabilities who may also have CVI as well as carefully evaluating these children’s functional vision when using the HFSW so appropriate accommodations can be provided.

### 4.4. Challenges of Using the HFSW

Both groups identified challenges associated with using the HFSW. Common issues included insufficient space and difficulties with transferring children. Professionals also highlighted challenges, such as moving over uneven surfaces and the time and staffing required to transfer children between wheelchairs and HFSWs. Previous studies provide support for these findings, identifying barriers like uneven surfaces, transfer difficulties, limited space, device instability, and the need for constant supervision [50,52]. These results emphasize the need to address design limitations of HFSWs. Many devices currently available require large, flat, and smooth areas, which are often unavailable in home and community settings. Additionally, transferring and positioning children in HFSWs places significant physical demands on adults, often requiring assistance of one or more caregivers. Constant supervision is also necessary for safety when a child is using a HFSW [50]. To mitigate these challenges, manufacturers, engineers, and a range of stakeholders—including professionals from multiple disciplines, school administrators, and parents—must collaborate to enhance the design of HFSWs and improve the environment, making them more accessible and user-friendly.

### 4.5. Clinical Implications

The responses from parents and professionals suggest that children with severe physical and communication disabilities were able to engage in various motor activities when using the HFSW. Additionally, the data indicate that HFSWs were effective in supporting a range of motor, educational, and communication goals. Its use can bring a range of benefits.

The benefits related to communication, language, and vision have important implications when providing AAC intervention to children with severe physical and communication disabilities. The upright, hands-free, self-initiated mobility provided by the HFSW may enhance these children’s communication and language ability by offering them new ways to express themselves and creating diverse language learning opportunities, as suggested by Luo and Blackstone [44].

The benefit related to vision is particularly significant for AAC because aided AAC tools rely heavily on vision [65,83,90,91]. The movement provided by the HFSW may improve vision in these children, the majority of whom have CVI. Additionally, self-initiated mobility supports the development of spatial perception, spatial memory, spatial cognition [36,39,80]. Better vision and spatial abilities can facilitate the development of language and communication as well as the use of aided AAC tools [44,87]. However, impaired vision can negatively impact a child’s self-initiated mobility so careful evaluation and ongoing monitoring of functional vision is important when utilizing the HFSW.

The findings of this exploratory survey also suggest several areas for improvement. Specifically, attention is needed to enhance the design and the implementation of HFSWs to ensure that children with severe physical and communication disabilities fully benefit from upright, hands-free, self-initiated mobility. Currently, HFSW designs do not accommodate the use of AAC tools and technologies, limiting access to language and communication—critical for developing peer relationships, self-determination and communication competence. Increased efforts to address design issues, educate and involve parents, coupled with improved collaboration among professionals, could amplify these benefits and optimize outcomes for these children.

#### 4.5.1. Providing More Information and Training to Parents

Family-centered practice is recommended by both researchers and professional organizations when working with these children [11,92]. Results from this study indicate that parents may have limited information about the use of the HFSW, particularly regarding goals or benefits. Professionals should prioritize explaining specific goals and benefits to parents, such as how the HFSW supports self-initiated mobility and how it can generate cascading benefits across motor, language, communication and vision domains. Parents should also receive structured training on how to effectively use the HFSW and be given opportunities to observe their children engaging in activities with the device. Parents may also need additional support to implement the HFSW at home or in community settings.

#### 4.5.2. Adopting Family-Centered Interprofessional Collaborative Practice

The current study identified the difference in perspectives between parents and professionals on the use of the HFSW and its benefits and presented some preliminary findings on differences among professionals from different disciplines. These differences highlight the importance of adopting a family-centered, interprofessional collaborative practice (IPCP), which offers a framework for parents and professionals from various fields to share knowledge and expertise to ensure the effective use of the HFSW. IPCP is recommended by various organizations including the American Speech–Language–Hearing Association (ASHA) [93], American Occupational Therapy Association (AOTA) [94], and American Physical Therapy Association (APTA) [95].

Challenges reported by participants underscore the design limitations of current HFSWs and AAC technologies. The design of the HFSW needs to be improved to resolve issues related to the environment and personnel. The future design of AAC technologies needs to better accommodate children with CVI and address issues related to accessing AAC when children are using the HFSW. Collaboration of multiple stakeholder groups is needed. The IPCP can provide a platform to bring together all stakeholders to address these obstacles and develop innovative solutions.

### 4.6. Limitations and Future Directions

This study is the first to explore the use of the HFSW with children with severe physical and communication disabilities. A few limitations must be noted. First, convenience sampling was used in the current study. Also, there was a regional concentration due to the outreach networks based in California. It may have reduced the representativeness of the sample, introduced selection bias, and shaped participants’ responses. Second, the sample size was relatively small, which may have limited the generalization of the study results. Third, there was an unequal number of participants in both groups, which may have caused difficulty in detecting some of the statistical differences between the two groups. Fourth, although both participant groups reported that children had severe physical and communication disabilities, the study did not consider certain characteristics within this population that could confound the interpretation of the results. These include the children’s intellectual abilities, access to professional services, and family socioeconomic status. Group differences may exist between the two groups. As a result, differences found between these two groups should be interpreted with caution. Fifth, although the participants were asked to report if the children used AAC, the study did not ask the context in which AAC was used. Specifically, did the children use AAC in wheelchairs and/or in HFSWs? This can be a confounding factor when interpreting the results. Next, questions on the surveys were mostly close-ended. The participants were provided “other” as a choice for most of the questions which was followed with a pop-up text box to write in answers in their own words. However, the predominant use of close-ended questions may have influenced participants’ responses and introduced bias. For example, benefits to communication might not have been reported if specific response options were absent. Additionally, social desirability bias may have contributed to overly positive perceptions of HFSW. Finally, the unequal representation of professionals from different disciplines constrained the analysis of differences across groups.

Future studies can utilize larger sample size with random sampling to obtain more diverse and representative samples. To further examine differences that may exist between parents and professionals and among professionals from different disciplines, equal sample sizes and ensure comparable samples should be implemented. Future studies can also prospectively stratify enrollment to address limitations such as the lack of role-specific denominators, the minimal demographic data collected from parents, and insufficient sample size to conduct reliable subgroup analyses by GMFCS level, CVI severity, or AAC modality.

Additional characteristics of children with severe physical and communication disabilities can be obtained to examine more closely on differences found between parents and professionals, such as intellectual ability, cultural and linguistic background, and the socioeconomic environment. Surveys with open-ended questions or qualitative research methods such as interviews and focus groups can be used to gain a deeper understanding of the perspectives of parents and professionals. In addition, all stakeholders including individuals with severe physical and communication disabilities who use the HFSW, engineers, manufacturers, and administrators, should be included.

The KidWalk and Rifton Pacer are the most commonly used HFSWs in this study, whereas David Hart orthosis walkers are the most extensively studied [50]. More studies on KidWalk and Rifton Pacer are needed to develop more comprehensive guidelines for professionals and parents. Research on how to optimize the HFSW and AAC design is also needed to address challenges identified in this study.

Studies are greatly needed to examine how children with severe physical and communication disabilities use AAC while in the HFSW. Although the current study did not ask participants whether or what AAC methods their children used in the HFSW, clinical observations have revealed the challenges of providing access to AAC tools or technologies in the HFSW due to the different positioning of the children and the limitations posed by the current design of AAC tools and HFSW. Access to AAC while in the HFSW is crucial, as these children need it to request assistance when maneuvering the equipment and to engage with others during exploration and social interactions. In the current study, although both groups identified the importance of “expanding opportunities to engage with peers”, future studies are needed to investigate the nature of communication barriers that children who use AAC and HFSW might face and how the design characteristics of the HFSW and AAC, particularly the speech-generating device (SGD), might be improved to better support these children.

Some specific design solutions that are needed include (a) how to mount a SGD on the HFSW; (b) how to access the SGD when the children are moving in an upright position; (c) how to add features to the HFSW so it can be used across different environments (e.g., different flooring, different room size, different surface grade, etc.); and (d) how to make the transfer more easily. A research study is currently being conducted at The Bridge School to examine how children with severe physical and communication disabilities communicate using AAC when they are participating in the regular school activities in the HFSW. The findings from this study can provide information related to intervention strategies, environment accommodations, as well as design considerations for the HFSW and the SGD.

Additionally, studies are needed to further examine the relationship between upright, hands-free, self-initiated mobility and language and communication development in children with severe physical and communication disabilities. Professionals in the current study reported some positive impact of upright, hands-free, self-initiated mobility on communication. Future studies can be conducted to examine how language and communication skills develop over time when these children have consistent access to upright, hands-free, self-initiated mobility. Studies can also examine how improved language and communication skills impact the self-initiated mobility in these children. The relationship between vision and upright, hands-free, self-initiated mobility in these children also needs further examinations. Longitudinal studies should be conducted to examine the long-term impacts of self-initiated mobility on various developmental domains in children with severe physical and communication disabilities.

Finally, the current study demonstrated differences in perspectives existing between parents and professionals regarding the use of AAC, the purpose for using the HFSW, and benefits of using the HFSW. Within the professionals, more ATs than OTs reported benefits related to communications. Although no statistical analysis was conducted due to the small sample size, this difference suggests that research is needed to examine the preservice and in-service training provided across different disciplines. Consideration of parent–professional collaboration and interprofessional collaboration in school settings is also an important research topic that requires further attention and investigation in order to optimize the use of HFSW and AAC with these children.

## 5. Conclusions

Upright, hands-free, self-initiated mobility—like walking—brings a range of benefits to children with typical development. Children with severe physical and communication disabilities can achieve this through the HFSW. Although more research is needed, this exploratory study demonstrates that the HFSW can help achieve a multitude of goals and benefits. However, the use of HFSW is still limited due to limited knowledge and design challenges. The use of AAC is not well understood when these children are in the HFSW. All stakeholders need to collaborate and share expertise so these children can access and experience upright, hands-free, self-initiated mobility, which can improve intervention outcomes in other key areas.

## Figures and Tables

**Table 1:** The number and percentage of professionals in each discipline.

Disciplines	Number of Participants	Percentage of the Total (N = 90)
Physical therapists	22	24.44
Speech–language pathologists	18	20.00
Special education teachers	13	14.44
Teachers of the visually impaired and/or orientation and mobility specialists	14	15.56
Assistive technologist	11	12.22
Occupational therapists	6	6.67
Paraprofessionals	6	6.67

**Table 2:** The purposes for using the HFSW.

Purposes	Parent Group (%)	Professional Group (%)	*z*	*p*	Cramér’s V
	(N = 21)	(N = 54)			
Achieve specific motor/mobility goals					
Provide independent mobility	76.19	94.44	−2.3	0.078	0.266
Improve hip development	47.62	40.74	0.54	0.677	0.062
Maintain bone mineral density	47.62	46.3	0.1	0.918	0.012
Reduce hip/knee ankle contracture	19.05	40.74	−1.77	0.146	0.204
Improve muscle strength	76.19	87.04	−1.15	0.411	0.133
Achieve cardiopulmonary exercise	28.57	53.7	−1.96	0.115	0.226
Reduce spasticity (hypertonus) through movement	28.57	33.33	−0.4	0.723	0.046
Achieve specific ADL/functional goals					
Improve digestive function	57.14	50	0.56	0.677	0.065
Encourage use of upper extremities for reaching/touching	61.9	70.37	−0.71	0.65	0.082
Encourage problem solving opportunities	47.62	72.22	−2.01	0.113	0.232
Increase independence and participation in daily life	52.38	83.33	−2.77	0.044 *	0.320
Participate in recess activities	42.86	70.37	−2.21	0.078	0.255
Encourage motivating activities	61.9	81.48	−1.78	0.146	0.206
Achieve educational goals					
Increase opportunities to participate with peers	66.67	88.89	−2.28	0.078	0.263
Access school environment	57.14	90.74	−3.35	0.013 *	0.387
Explore surroundings	85.71	90.74	−0.63	0.672	0.073
Move to specific location (e.g., retrieve lunch bag)	38.1	77.78	−3.27	0.012 *	0.378
Move close to see objects and people	57.14	85.19	−2.6	0.053	0.300
Access recess activities such as running, jumping, and playground games	38.1	66.67	−2.26	0.078	0.261
Participate in inclusive physical education	57.14	62.96	−0.46	0.703	0.053
Increase activity level	71.43	85.19	−1.37	0.3	0.158
I am not sure	0	5.56	−1.1	0.415	0.127
Other	9.52	3.7	1.01	0.451	0.117

*Note*: * Statistically significant at *p* < 0.05.

**Table 3:** The benefits observed when using the HFSW.

Benefits	Parent Group (%)	Professional Group (%)	*z*	*p*	Cramér’s V
	(N = 21)	(N = 64)			
Physical benefits					
Achieves a means for exercising and being physical active	71.43	85.94	−1.51	0.176	0.164
Increase opportunities for self-initiated mobility	61.9	93.75	−3.64	0.007 *	0.395
Improves motor control (e.g., stepping, reaching, head and trunk)	66.67	76.56	−0.9	0.386	0.098
Encourages use of arms/hands for reaching and exploring	52.38	78.13	−2.27	0.053	0.246
Enables a child to move close to view and touch objects and people	52.38	81.25	−2.62	0.037 *	0.284
Enables a child to move towards a desired place or move away from an undesirable place or event	57.14	84.38	−2.6	0.037 *	0.282
Acquires new motor skills like jumping, wiggling, running, spinning	19.05	50	−2.49	0.037 *	0.270
Improves muscle strength	57.14	78.13	−1.88	0.099	0.204
Improves bone mineral density	23.81	50	−2.1	0.073	0.228
Provides a change in position	71.43	85.94	−1.51	0.176	0.164
Assists with gastroenterology issues and regularity	42.86	59.38	−1.32	0.216	0.143
Social/Emotional/Participation benefits					
Brings enjoyment: Child smiles, laughs, wants to use the support walker	85.71	85.94	−0.03	0.98	0.003
Increases independence and access to surroundings (e.g., opens/closes drawers, reaches for objects, moves to favorite location, plays at peer height)	71.43	84.38	−1.32	0.216	0.143
Increases peer/sibling/family interaction	52.38	68.75	−1.36	0.216	0.148
Benefits in other key developmental areas					
Improves communication (e.g., points with arm/hand to a desired location, moves close to a peer to interact, moves away from an undesired event, moves self to seek a needed object like food/drink, moves upon request, answers a question (e.g., “What’s next?”, “Where is the group?”) by moving to that location)	38.1	68.75	−2.5	0.037 *	0.271
Increases verbalization/vocalizations while standing and moving in a hands-free support walker	33.33	67.19	−2.74	0.037 *	0.297
Demonstrates more eye contact with people or objects	33.33	62.5	−2.33	0.050 *	0.253
Uses movement in a hands-free support walker to attain or sustain vision before looking at a visual stimulus (e.g., wiggling, jumping, bouncing)	33.33	53.13	−1.58	0.176	0.171
Demonstrates curiosity and problem solving (e.g., interacting with an object to learn how it works, such as turning on a water fountain or opening a door handle, finding and exploring a new place or objects)	42.86	75	−2.72	0.037 *	0.295
Is more attentive and focused after moving in a hands-free support walker	33.33	45.31	−0.96	0.367	0.104
Uses vision to kick a ball, look for feet, reach for a favorite object, move toward/away from a person or an activity, explore the environment	33.33	59.38	−2.07	0.073	0.225
Other options					
No benefits have been observed	9.52	0	2.5	0.037 *	0.271
Other	14.29	3.13	1.89	0.099	0.205

*Note:* * Statistically significant at *p* < 0.05.

**Table 4:** Difficulties encountered when using a HFSW.

Problems	Parent Group (%)	Professional Group (%)	*z*	*p*	Cramér’s V
	(N = 21)	(N = 62)			
Not enough space indoors to use it functionally	61.9	62.9	−0.08	0.956	0.009
Difficulty for the child or the client to move over uneven surfaces (e.g., cracks in sidewalk, over carpet, thresholds)	57.14	75.81	−1.63	0.464	0.179
Transfers in/out of the walker are difficulty	57.14	56.45	0.06	0.956	0.007
Time (and staff) required to transfer the child or the client in/out of the walker	38.1	62.9	−1.98	0.427	0.217
Finding time for the child or the client to use the walker	42.86	32.26	0.88	0.585	0.097
Difficulty adjusting it (for multiple users)	28.57	16.13	1.25	0.476	0.137
Therapist or care provider’s belief that using the walker will not support the child’s or the client’s therapy goals	14.29	9.68	0.59	0.716	0.065
Musculoskeletal issues (e.g., spasticity, muscle tightening, muscle fatigue, etc.)	23.81	33.87	−0.86	0.585	0.094
Other	28.57	16.13	1.25	0.476	0.137

## Data Availability

The datasets generated and/or analyzed during the current study are available from the corresponding author on reasonable request.

## Supplementary Materials

The following supporting information can be downloaded at: https://www.mdpi.com/article/10.3390/children12081024/s1, Supplementary Survey A: Initial Survey; Supplementary Survey B: Survey for Parents/Care Providers; Supplementary Survey C: Survey for Professionals/Paraprofessionals; Supplementary Table S1: Comparison of Types of Motor Activities Observed by Parent and Professional group. Table S2: Comparison of Types of Motor Activities Observed by the Parent and Professional Groups.

## Abbreviations

The following abbreviations are used in this manuscript:

HFSW	Hands-free support walker
AAC	Augmentative and alternative communication
CVI	Cortical visual impairment
