# Supplementary material for: Surveying the Perspectives of Parents and Professionals on Providing Upright, Hands-Free, Self-Initiated Mobility to Children with Severe Physical and Communication Disabilities
Source: Children (Basel). 2025 Aug 4;12(8):1024. doi: 10.3390/children12081024 (PMC12384451; doi:10.3390/children12081024)
Supplement: Supplementary file 1 [file children-12-01024-s001.zip › children-3725295-supplementary.pdf]

Supplemental Materials

Providing Mobility to Children with Severe Physical and Communication Disabilities

Supplementary Survey A: Initial Survey

1. Are you a parent/care provider or a professional/paraprofessional? (Single choice response): Parent/care provider; Professional/paraprofessional
2. Have you worked with children with complex speech and physical impairments? (Single choice response): Yes; No
3. Are you currently working with and/or have you worked with children who use hands-free support walkers within the last 10 years? A hands-free support walker, also referred to as a gait trainer, is designed with a seat, upper body supports and possibly a headrest, allowing the user to stand and move with hands-free mobility to explore, reach and interact (e.g.., KidWalk, Pacer, Grillo, etc.). (Single choice response): Yes; No
4. For what reason(s) might your clients not be using a hands-free support walker? (Multiple choices response): Funding issues; The client outgrew the walker; The clients couldn't move it due to spasticity and leg stiffness; The client could not maneuver the walker over various outdoor/indoor surfaces; It was too difficult for staff to transfer the client; The parent did not want to use it; No reason identified; Other, please specify.
5. Does your child have complex speech and physical impairments? (Single choice response): Yes; No
6. Is your child currently using or in the past has used a hands-free support walker? A hands-free support walker, also referred to as a gait trainer, provides mobility to a child with a seat, upper body support and possibly a headrest, but does not require the child to hold onto the walker to steer it (e.g., KidWalk; ProneWalker, Pacer, Grillo, Mustang, etc.). (Single choice response): Yes; No
7. For what reason(s) might your child not be using a hands-free support walker? (Multiple choices response): Funding issues; Therapists did not recommend it; My child couldn't move it due to spasticity and leg stiffness; My child did not enjoy it; Not enough space at home; Other, please specify.
8. In general, do you believe that children with complex speech and physical impairments can benefit from self-directed mobility experiences provided by hands-free support walkers? (Single choice response): Yes; No; I am not sure.
9. which of the following areas do you think the hands-free support walker can have a positive impact on? Select all that apply. (Multiple choices responses)
    a. Physical (e.g., being physically active, increasing independent mobility)
    b. Social (e.g., increasing interaction with peers)
    c. Communication (e.g., increasing vocalization, using movement to answer questions)
    d. Self-determination (e.g., signaling preferences by going toward/away from something or someone)
    e. Participation (e.g., increasing participation in sports and recess activities)
    f. Cognitive (e.g., providing problem solving opportunities, manipulating objects to learn how they work)
    g. Emotional (e.g., bringing enjoyment and sense of independence)
    h. Spatial (e.g., navigating through space)
    i. Visual (e.g., using vision to reach and grab objects)
    j. Other, please specify

Supplementary Survey B: Survey for Parents/Care Providers

<u>Parent/Care Provider Information on Child and Program</u>

1. In what state do you live? (Single choice response): List of 50 States; International
2. How old is your child? (Text response): ______________________________ ____________________________________
3. Is your child currently participating in an educational program? (Single choice response): Yes; No
4. Please indicate the type of the educational program. (Single choice response): Preschool; Regular education class; Special education class; Private school; Home schooling; Other, please specify
5. What therapy services does your child receive at school? Select all that apply. (Multiple choice response): None; Physical therapy; Occupational therapy; Speech therapy; Special education services; Assistive technology; Orientation and mobility services; Low vision therapy; Other, please specify
6. What therapy services does your child receive when NOT at school? Select all that apply. (Multiple choices response): None; Physical therapy; Occupational therapy; Alternative practitioner, please specify; Speech therapy; Orientation and mobility services, Low vision therapy; Home-based special education services; Other, please specify
7. Please indicate the medical conditions of your child from the list below. (Multiple choices response): Cerebral Palsy; Chromosomal Abnormalities; Spina Bifida; Muscular Dystrophy; Rett Syndrome; Epilepsy; Spinal Cord Injury; Developmental Delay; Complex Communication Needs; Other, please specify
8. What type of Cerebral Palsy does your child have? (Multiple choice response): Spastic Quadriplegia; Spastic Diplegia; Spastic Hemiplegia; Dyskinetic (athetosis or dystonia); Ataxic; Hypotonic; I am not sure.
9. Which medical interventions has your child had past the age of 3? (Multiple choices response): Botox; Orthopedic surgery; Dorsal root rhizotomy; Oral baclofen; Baclofen pump; Other, please specify
10. Considering the Gross Motor Function Classification System (GMFCS), at which level do you know or think your child is at currently? (Single choice response): Level I; Level II; Level III; Level IV; Level V; I am not sure.
11. Does your child wear Ankle Foot Orthoses (AFOs), which start at the toes and go to the upper calf)? (Single choice response): Yes; No
12. Are they hinged at the ankle? (Single choice response): Yes; No
13. Are they rigid and solid with no movement? (Single choice response): Yes; No
14. What adaptive and mobility equipment does your child use? Select all that apply. (Multiple choices responses):
    a. Adaptive Equipment: Activity chair; Supine stander/standing frame where a child's back rests on the pads; Prone stander where a child's abdomen rests against the pads; Stander; Stander with reachable wheels
    b. Manual Mobility Equipment: Stroller; Non-self-propelling manual wheelchair (dependent base with small wheels); Self-propelling manual wheelchair; Self-propelling manual stand-up wheelchair
    c. Powered Equipment: Adapted battery powered toy car/alternatives; Explorer Mini (for children aged 0 to 3); Power wheelchair with joystick; Power wheelchair with standing feature; Power wheelchair with lowering to the floor feature; Power wheelchair with alternative controls like head array
    d. Other, please specify
15. Does your child use a hand-held push walker? A hand-held push walker provides no body supports. A child can stand and hold onto handles of the walker to push or pull it along. (Single choice response): Yes; No; I am not sure.
16. What are the purposes for using the hand-held push walker? (Multiple choices response):
    a. Achieve specific motor/mobility goals: Provide independent mobility; Improve hip development; Maintain bone mineral density; Reduce hip/knee ankle contractures; Improve muscle strength; Achieve cardiopulmonary exercise; Reduce spasticity (hypertonus) through movement
    b. Achieve specific ADL/functional goals: Improve digestive function; Encourage use of upper extremities for reaching/touching; Encourage problem solving opportunities; Increase independence and participation in daily life; Participate in recess activities; Encourage motivating activities
    c. Achieve educational goals: Increase opportunities to participate with peers; Accessing school environment; Exploring surroundings; Move to a specific location (e.g., retrieve lunch bag);

Move close to see objects and people; Access recess activities such as running, jumping, and playground games; Participate in inclusive physical education; Increase activity level

d. Other, please specify

e. I am not sure.

17. Does your child use augmentative and alternative communication systems (AAC)? (Single choice response): Yes; No; I am not sure
18. please indicate AAC methods your child uses. Select all that apply. (Multiple choice response)
    a. Body-based/no-tech methods: Gestures (e.g., head nod – yes, head shake- no, reaching, pointing); Manual signs (e.g., drink, more); Vocalization (e.g., laughing, squealing, babbling); Facial expressions (e.g., happy, anger, sad, questioning); speech (e.g., recognizable words/word approximations); Body postures (e.g., head down/up to indicate interest); Movement toward person/object (e.g., going toward or away)
    b. Non-electronic/low-tech methods: Photos/symbols placed strategically in environment (e.g., kitchen, bathroom, etc.); Communication board(s) with symbols/photos/text; Communication book(s) with symbols/photos/text; Commercial AAC product (e.g., PODD, PEC, etc.)
    c. Electronic/high-tech methods: Simple AAC device with digitized speech output; Complex AAC device with synthesized speech output; Tablet with communication app; Smart phone with communication app; Computer with software; Email; Social media
    d. Other, please specify
19. Does your child have a visual impairment? (Single choice response): Yes; No; I am not sure
20. Please indicate types of visual impairments. (Multiple choices responses): Cortical visual impairment (CVI); Optic nerve disorder; Retinal disorder; Refractive errors (e.g., myopia, hyperopia, astigmatism); Strabismus including exotropia or esotropia; Other, please specify; Unknown
21. Does your child wear glasses? (Single choice response): Yes; No

<u>Parent/Care provider Use of and Opinions on Hands-Free Support Walkers</u>

22. Which types of hands-free support walkers does your child use? Select all that apply. (Multiple choices response): KidWalk; ProneWalk; Winnipeg/Pommel Walker; Rifton Pacer; My Way Leckey; Buddy Roamer; Grillo Ormesa; Dynamico Ormesa; Mustang R82; Mini Walk; Meywalk; Other, please specify
23. How old was your child when he/she first used a hands-free support walker? (Text response): ______________________
24. How many years has your child used the hands-free support walker? (Single choice response): 1-3 years; 4-6 years; 7-10 years; >11 years
25. What can your child do in the hands-free support walker? (Multiple choices response): Pushes backwards; Takes more than 5 steps forwards; Turns either direction; Runs; Spins in circles; Jumps; Wiggles; Kicks ball; Throws ball; Moves close to look at objects or people; Moves close to reach and touch objects or people; Other, please specify
26. Please indicate the average amount of time per day your child is in the hands-free support walker at home. (Single choice response): Less than 1 hour; 1-2 hours; 2-3 hours; 3-4 hours; 4-5 hours; More than 5 hours
27. Where does your child use the hands-free support walker? (Multiple choices response): School; Clinical setting; At home; In the community; Other, please specify
28. What are the purposes for using the hands-free support walker? (Multiple choices response):
    a. Achieve specific motor/mobility goals: Provide independent mobility; Improve hip development; Maintain bone mineral density; Reduce hip/knee ankle contractures; Improve muscle strength; Achieve cardiopulmonary exercise; Reduce spasticity (hypertonus) through movement
    b. Achieve specific ADL/functional goals: Improve digestive function; Encourage use of upper extremities for reaching/touching; Encourage problem solving opportunities; Increase independence and participation in daily life; Participate in recess activities; Encourage motivating activities
    c. Achieve educational goals: Increase opportunities to participate with peers; Accessing school environment; Exploring surroundings; Move to a specific location (e.g., retrieve lunch bag); Move close to see objects and people; Access recess activities such as running, jumping, and playground games; Participate in inclusive physical education; Increase activity level
    d. Other, please specify

e. I am not sure.

29. What are the top THREE most frequent activities your child participates in when using the hands-free support walker? (Multiple choices response): Therapy; Inclusive Physical Education; Recess; Academic lessons; Mobility in the classroom; Field trips; Play; Sports; Community activities (e.g., shopping, playing at the park); Home activities (e.g., chores, interacting with siblings); Other, please specify
30. What are the benefits you have observed from your child when using the hands-free support walker? Select all that apply. (Multiple choices response).
    a. Physical Benefits
        i. Achieves a means for exercising and being physically active
        ii. Increases opportunities for self-directed mobility
        iii. Improves motor control (e.g., stepping, reaching, head and trunk)
        iv. Encourages use of arms/hands for reaching and exploring
        v. Enables a child to move close to view and touch objects and people
        vi. Enables a child to move towards a desired place or move away from an undesirable place or event
        vii. Acquires new motor skills like jumping, wiggling, running, spinning
        viii. Improves muscle strength
        ix. improves bone mineral density
        x. Provides a change of position
        xi. Assists with gastroenterology issues and regularity
    b. Social/Emotional/Participation
        i. Brings enjoyment: Child smiles, laughs, wants to use the support walker
        ii. Increases independence and access to surroundings (e.g., opens/closes drawers, reaches for objects, moves to favorite location, plays at peer height)
        iii. Increases peer/sibling/family interaction
    c. Other Key Developmental Areas
        i. Improves communication (e.g., points with arm/hand to a desired location, moves close to a peer to interact, moves away from an undesired event, moves self to seek a needed object like food/drink, moves upon request, answers a question (e.g., "What's next?", "Where is the group?") by moving to that location)
        ii. Increases verbalization/vocalizations while standing and moving in a hands-free support walker
        iii. Demonstrates more eye contact with people or objects
        iv. Uses movement in a hands-free support walker to attain or sustain vision before looking at a visual stimulus (e.g., wiggling, jumping, bouncing)
        v. Demonstrates curiosity and problem solving (e.g., interacting with an object to learn how it works, such as turning on a water fountain or opening a door handle; finding and exploring a new place or objects)
        vi. Is more attentive and focused after moving in a hands-free support walker
        vii. Uses vision to kick a ball, look at feet, reach for a favorite object, move toward/away from a person, activity, explore the environment
    d. Other options
        i. No benefits have been observed
        ii. Other, please specify
31. What difficulties have you encountered when using a hands-free support walker? Select all that apply (Multiple choices response).

    a. Not enough space indoors to use it functionally
    b. Difficult for the child to move over uneven surfaces (e.g., cracks in sidewalk, over carpet, thresholds)
    c. Transfers in/out of the walker are difficult
    d. Time required to transfer the child in/out of walker
    e. Finding time for the child to use the walker
    f. Difficult to adjust

   g. Therapist or care provider’s belief that using a hands-free support walker will not support the child’s therapy goals. Please specify “who”.
   h. Musculoskeletal issues (e.g., spasticity, muscle tightening, muscle fatigue, etc.)
   i. Other, please specify
      a. If “Therapist or care provider’s belief that using a hands-free support walker will not support the child’s therapy goal” is selected, please specify “who”. (Single choice response): Therapist; Parent; Both

32. How is your child’s hands-free support walker funded? (Multiple choices response): Private insurance; Medicaid; School district; Self pay; Donation; Other, please specify
33. Would you like to provide us with your contact information so you can stay informed of this project or other projects? (Single choice response): Yes; No

Supplementary Survey C: Survey for Professionals/Paraprofessionals

Information about Professionals/Paraprofessionals and Clients

1. In what state do you work? (Single choice response): List of 50 States; International
2. What is your profession? Select all that apply. (Multiple choice response): Occupational Therapist (OT); Physical Therapist (PT); Speech-Language Pathologist (SLP); OT/PT/SLP Assistant; General Education Teacher; Special Education Teacher; Teacher of the Visually Impaired (TVI); Orientation & Mobility Specialist (O&M); Assistive Technology Professional; Instructional Assistant/Paraprofessionals; Administrator; Supervising Therapist; Other, please specify
3. How many years have you been a practicing professional? (Single choice response): 1-5 years; 6-10 years; >11 years
4. Where do you provide services currently? Select all that apply. (Multiple choice response):
    a. In schools: Public school district, multiple sites; Public school district, single site; Private school; School for the blind; Special education school; Charter school; Other, please specify
    b. In clinical settings: Hospital; Rehabilitation center; Community therapy clinic (e.g., California Children's Services); Private practice clinic; State agencies (e.g., Regional Centers, SELPA in CA); Other, please specify
    c. In home or community: Client's home; Community center; Library; Other, please specify
5. What age groups do you currently serve? (Multiple choices response): Birth to 3 years; 4-5 years; 6-12 years; 13-22 years; > 22 years
6. What adaptive equipment (devices) do you evaluate and recommend, in collaboration with other team members, as appropriate? (Multiple choices response): None; Support walkers (Gait Trainers); Stander; Manual wheelchair; Power wheelchair; Stroller; Activity chair; Computer technology; Augmentative and alternative communication tools/technologies; Speech-generating devices; Adaptive toys; Switches; Low vision aids; Other, please specify
7. Are you currently working with children who use hands-free support walkers? (Single choice response): Yes; No
8. Please indicate the medical conditions of your clients from the list below. (Multiple choices response): Cerebral Palsy; Chromosomal Abnormalities; Spina Bifida; Muscular Dystrophy; Rett Syndrome; Epilepsy; Spinal Cord Injury; Developmental Delay; Complex communication needs; Other, please specify
9. What type of Cerebral Palsy do your clients have? (Multiple choice response): Spastic Quadriplegia; Spastic Diplegia; Spastic Hemiplegia; Dyskinetic (athetosis or dystonia); Ataxic; Hypotonic; I am not sure.
10. Considering the Gross Motor Function Classification System (GMFCS), please indicate the levels of your clients. (Multiple choice response): Level I; Level II; Level III: Level IV; Level V; I am not sure.
11. What adaptive and mobility equipment do your clients use? Select all that apply. (Multiple choices responses):
    a. Adaptive Equipment: Activity chair; Supine stander/standing frame where a child's back rests on the pads; Prone stander where a child's abdomen rests against the pads; Stander; Stander with reachable wheels
    b. Manual Mobility Equipment: Stroller; Non-self-propelling manual wheelchair (dependent base with small wheels); Self-propelling manual wheelchair; Self-propelling manual stand-up wheelchair
    c. Powered Equipment: Adapted battery powered toy car/alternatives; Explorer Mini (for children aged 0 to 3); Power wheelchair with joystick; Power wheelchair with standing feature; Power wheelchair with lowering to the floor feature; Power wheelchair with alternative controls like head array
    d. Other, please specify
12. Do any of your clients use hand-held push walkers? A hand-held push walker provides no body supports. A child can stand and hold onto handles of the walker to push or pull it along. (Single choice response): Yes; No; I am not sure.
13. What are the purposes for using the hand-held push walker? Select all that apply. (Multiple choices response):
    a. Achieve specific motor/mobility goals: Provide independent mobility; Improve hip development; Maintain bone mineral density; Reduce hip/knee ankle contractures; Improve muscle strength; Achieve cardiopulmonary exercise; Reduce spasticity (hypertonus) through movement

   b. Achieve specific ADL/functional goals: Improve digestive function; Encourage use of upper extremities for reaching/touching; Encourage problem solving opportunities; Increase independence and participation in daily life; Participate in recess activities; Encourage motivating activities
   c. Achieve educational goals: Increase opportunities to participate with peers; Accessing school environment; Exploring surroundings; Move to a specific location (e.g., retrieve lunch bag); Move close to see objects and people; Access recess activities such as running, jumping, and playground games; Participate in inclusive physical education; Increase activity level
   d. Other, please specify
   e. I am not sure.
14. Do your clients use augmentative and alternative communication systems (AAC)? (Single choice response): Yes; No; I am not sure.
15. Please indicate AAC methods your clients use. Select all that apply. (Multiple choice response)
   a. Body-based/no-tech methods: Gestures (e.g., head nod – yes, head shake- no, reaching, pointing); Manual signs (e.g., drink, more); Vocalization (e.g., laughing, squealing, babbling); Facial expressions (e.g., happy, anger, sad, questioning); Speech (e.g., recognizable words/word approximations); Body postures (e.g., head down/up to indicate interest); Movement toward person/object (e.g., going toward or away)
   b. Non-electronic/low-tech methods: Photos/symbols placed strategically in environment (e.g., kitchen, bathroom, etc.); communication board(s) with symbols/photos/text; communication book(s) with symbols/photos/text; commercial AAC product (e.g., PODD, PEC, etc.)
   c. Electronic/high-tech methods: Simple AAC device with digitized speech output; Complex AAC device with synthesized speech output; Tablet with communication app; Smart phone with communication app; Computer with software; Email; Social media
   d. Other, please specify
16. Do your clients have a visual impairment? (Single choice response): Yes; No; I am not sure.
17. Please indicate types of visual impairments that your clients have. (Multiple choices response): Cortical visual impairment (CVI); Optic nerve disorder; Retinal disorder; Refractive errors (e.g., myopia, hyperopia, astigmatism); Strabismus including exotropia or esotropia; Other, please specify; Unknown
18. Do your clients wear glasses? (Single choice response): Yes; No

<u>Professional/Paraprofessional Use of Hands-Free Support Walkers</u>

19. Which types of hands-free support walkers do your clients currently use? Select all that apply. (Multiple choices response): KidWalk; ProneWalk; Winnipeg/Pommel Walker; Rifton Pacer; My Way Leckey; Buddy Roamer; Grillo Ormesa; Dynamico Ormesa; Mustang R82; Mini Walk; Meywalk; Other, please specify.
20. Over the past 10 years, how many of your clients have used hands-free support walkers? (Single choice response): 1-5; 6-10; 11-20; more than 20
21. What are the GMFCS levels of your clients who currently use hands-free support walkers? (Multiple choices response): Level I; Level II; Level III; Level IV; Level V; I am not sure.
22. What percentage of your clients who currently use hands-free support walkers wear Ankle Foot Orthoses (AFOs), which start at the toes and go to the upper calf? (Slider scale response): 0-100%
23. What percentage of AFOs are hinged at the ankle? (Slider scale response): 0-100%
24. What percentage of AFOs are rigid and solid with no movement? (Slider scale response): 0-100%
25. What motor activities have you observed your clients achieve in hands-free support walkers? (Multiple choices response): Pushes backwards; Takes more than 5 steps forwards; Turns either direction; Runs; Spins in circles; Jumps; Wiggles; Kicks ball; Throws ball; Moves close to look at objects or people; Moves close to reach and touch objects or people; Other, please specify
26. Please indicate the average amount of time per day your clients are in hands-free support walkers. (Multiple choices response): Less than 1 hour; 1-2 hours; 2-3 hours; 3-4 hours; 4-5 hours; More than 5 hours
27. Where do your clients use hands-free support walkers? (Multiple choices response): School; Clinical setting; At home; In the community; Other, please specify
28. What are the purposes for using hands-free support walkers? Select all that apply. (Multiple choices response):

a. Achieve specific motor/mobility goals: Provide independent mobility; Improve hip development; Maintain bone mineral density; Reduce hip/knee ankle contractures; Improve muscle strength; Achieve cardiopulmonary exercise; Reduce spasticity (hypertonus) through movement
b. Achieve specific ADL/functional goals: Improve digestive function; Encourage use of upper extremities for reaching/touching; Encourage problem solving opportunities; Increase independence and participation in daily life; Participate in recess activities; Encourage motivating activities
c. Achieve educational goals: Increase opportunities to participate with peers; Accessing school environment; Exploring surroundings; Move to a specific location (e.g., retrieve lunch bag); Move close to see objects and people; Access recess activities such as running, jumping, and playground games; Participate in inclusive physical education; Increase activity level
d. Other, please specify
e. I am not sure.

29. What are the top THREE most frequent activities your clients participate in when using hands-free support walkers? (Multiple choices response): Therapy; Inclusive Physical Education; Recess; Academic lessons; Mobility in the classroom; Field trips; Play; Sports; Community activities (e.g., shopping, playing at the park); Home activities (e.g., chores, interacting with siblings); Other, please specify

<u>Professional/Paraprofessional Opinions on Hands-Free Support Walkers</u>

30. What are the benefits you have observed from your clients when using hands-free support walkers? Select all that apply. (Multiple choices response).
    a. Physical Benefits
        i. Achieves a means for exercising and being physically active
        ii. Increases opportunities for self-directed mobility
        iii. Improves motor control (e.g., stepping, reaching, head and trunk)
        iv. Encourages use of arms/hands for reaching and exploring
        v. Enables a child to move close to view and touch objects and people
        vi. Enables a child to move towards a desired place or move away from an undesirable place or event
        vii. Acquires new motor skills like jumping, wiggling, running, spinning
        viii. Improves muscle strength
        ix. improves bone mineral density
        x. Provides a change of position
        xi. Assists with gastroenterology issues and regularity
    b. Social/Emotional/Participation
        i. Brings enjoyment: Child smiles, laughs, wants to use the support walker
        ii. Increases independence and access to surroundings (e.g., opens/closes drawers, reaches for objects, moves to favorite location, plays at peer height)
        iii. Increases peer/sibling/family interaction
    c. Other key developmental areas
        i. Improves communication (e.g., points with arm/hand to a desired location, moves close to a peer to interact, moves away from an undesired event, moves self to seek a needed object like food/drink, moves upon request, answers a question (e.g., "What's next?", "Where is the group?") by moving to that location)
        ii. Increases verbalization/vocalizations while standing and moving in a hands-free support walker
        iii. Demonstrates more eye contact with people or objects
        iv. Uses movement in a hands-free support walker to attain or sustain vision before looking at a visual stimulus (e.g., wiggling, jumping, bouncing)
        v. Demonstrates curiosity and problem solving (e.g., interacting with an object to learn how it works, such as turning on a water fountain or opening a door handle; finding and exploring a new place or objects)
        vi. Is more attentive and focused after moving in a hands-free support walker

        vii. Uses vision to kick a ball, look at feet, reach for a favorite object, move toward/away from a person or an activity, explore the environment
    d. Other options
        i. No benefits have been observed
        ii. Other, please specify

31. What difficulties have you encountered when using a hands-free support walker? Select all that apply. (Multiple choices response).
    a. Not enough space indoors to use it functionally
    b. Difficult for the client to move over uneven surfaces (e.g., cracks in sidewalk, over carpet, thresholds)
    c. Transfers in/out of the walker are difficult
    d. Time and staff required to transfer the client in/out of the walker
    e. Finding time for the client to use the walker
    f. Difficulty adjusting it for multiple users
    g. Therapist or care provider's belief that using the walker will not support the client's therapy goal
    h. Musculoskeletal issues (e.g., spasticity, muscle tightening, muscle fatigue, etc.)
    i. Other, please specify
        a. If "Therapist or care provider's belief that using a hands-free support walker will not support the client's therapy goal" is selected, please specify "who". (Single choice response): Therapist; Parent; Both
32. How are hands-free support walkers funded? (Multiple choices response): Private insurance; Medicaid; School district; Self pay; Donation; Other
33. Would you like to provide us with your contact information so you can stay informed of this project or other projects? (Single choice response): Yes; No

Supplementary Table S1: The type of the HFSW devices used.

Name of the HFSW	Parent Group (N=22)	Professional Group (N=53)
KidWalk1	54.55	75.47
ProneWalk1	9.09	16.98
Winnipeg/Pommel Walker2	0.00	0.00
Rifton Pacer3	18.18	69.81
My Way Leckey4	0.00	15.09
Buddy Roamer5	0.00	3.77
Grillo Ormesa6	4.55	30.19
Dynamico Ormesa6	0.00	3.77
Mustang R827	9.09	37.74
Mini Walk8	0.00	5.66
MeyWalk8	0.00	11.32
Other	31.82	3.77

[1]manufactured by Prime Engineering, Fresno CA; [2]manufactured by Rehabilitation Center for Children Sscy Center, Manitoba, Canada; [3]manufactured by Rifton Equipment, Rifton NY; [4]manufactured by Leckey, Lisburn, United Kingdom; [5]manufactured by the Moorings Mediquip, Ballymena, United Kingdom; [6]manufactured by Ormesa, Foligno, Italy; [7]manufactured by Etac, headquartered in Sweden; [8]manufactured by VELA, Anlborg SV, Denmark

Supplementary Table S2: Comparison of Types of Motor Activities Observed by the Parent and Professional Groups

Motor Activities Observed	Parent Group (%) (N=19)	Professional Group (%) (N=52)	*z*	*p*	Cramér's V
Pushes backwards	42.11	92.31	4.59	<.0001*	0.545
Takes more than 5 steps forward	78.95	86.54	0.78	0.4733	0.093
Turns either direction	63.16	78.85	1.35	0.2563	0.160
Runs	21.05	28.85	0.66	0.511	0.078
Spins in circles	21.05	53.85	2.46	.0334*	0.292
Jumps	5.26	25	1.85	0.1284	0.220
Wiggles	21.05	44.23	1.78	0.1284	0.211
Kicks ball	31.58	67.31	2.7	.028*	0.320
Throws ball	10.53	42.31	2.51	.0334*	0.298
Moves close to look at objects or people	68.42	82.69	1.3	0.2563	0.154
Moves close to reach and touch objects or people	57.89	88.46	2.87	.0252*	0.341
Other	15.79	7.69	-1.01	0.373	0.120
